# Monte Carlo simulations of cefepime in children receiving continuous kidney replacement therapy support continuous infusions for target attainment

**DOI:** 10.1186/s40560-024-00752-0

**Published:** 2024-10-08

**Authors:** H. Rhodes Hambrick, Nieko Punt, Kathryn Pavia, Tomoyuki Mizuno, Stuart L. Goldstein, Sonya Tang Girdwood

**Affiliations:** 1 Division of Nephrology and Hypertension, Cincinnati Children’s Hospital Medical Center (CCHMC), 3333 Burnet Avenue, Cincinnati, OH 45229 USA; 2 Division of Translational and Clinical Pharmacology, CCHMC, 3333 Burnet Avenue, Cincinnati, OH 45229 USA; 3 Division of Nephrology, Department of Pediatrics, Ann & Robert H. Lurie Children’s Hospital of Chicago, 225 E Chicago Ave, Chicago, IL 60611 USA; 4 Medimatics, Praaglaan 131, 6229 HR Maastricht, Netherlands; 5 Department of Clinical Pharmacy and Pharmacology, University of Groningen, University Medical Center Groningen, Hanzeplein 1, 9713 GZ Groningen, Netherlands; 6 Division of Critical Care Medicine, CCHMC, 3333 Burnet Avenue, Cincinnati, OH 45229 USA; 7 Department of Pediatric Critical Care, Children’s Mercy Kansas City, 2401 Gillham Rd, Kansas City, MO 64108 USA; 8 Department of Pediatrics, University of Cincinnati College of Medicine, 3230 Eden Avenue, Cincinnati, OH 45229 USA; 9 Center for Acute Care Nephrology, CCHMC, 3333 Burnet Avenue, Cincinnati, OH 45229 USA; 10 Division of Hospital Medicine, CCHMC, 3333 Burnet Avenue, Cincinnati, OH 45229 USA; 11 875 N Michigan Ave, Suite 1500, Chicago, IL 60611 USA

**Keywords:** CKRT, Beta-lactam pharmacokinetics, Pediatric acute kidney injury

## Abstract

**Background:**

Sepsis is a leading cause of acute kidney injury requiring continuous kidney replacement therapy (CKRT) and CKRT can alter drug pharmacokinetics (PK). Cefepime is used commonly in critically ill children and is cleared by CKRT, yet data regarding cefepime PK and pharmacodynamic (PD) target attainment in children receiving CKRT are scarce, so we performed Monte Carlo simulations (MCS) of cefepime dosing strategies in children receiving CKRT.

**Methods:**

We developed a CKRT “module” in the precision dosing software Edsim++. The module was added into a pediatric cefepime PK model. 1000-fold MCS were performed using six dosing strategies in patients aged 2–25 years and ≥ 10 kg with differing residual kidney function (estimated glomerular filtration rate of 5 vs 30 mL/min/1.73 m^2^), CKRT prescriptions, (standard-dose total effluent flow of 2500 mL/h/1.73 m^2^ vs high-dose of 8000 mL/h/1.73 m^2^), and fluid accumulation (0–30%). Probability of target attainment (PTA) was defined by percentage of patients with free concentrations exceeding bacterial minimum inhibitory concentration (MIC) for 100% of the dosing interval (100% *f*T > 1xMIC) and 4xMIC using an MIC of 8 mg/L for *Pseudomonas aeruginosa*.

**Results:**

Assuming standard-dose dialysis and minimal kidney function, > 90% PTA was achieved for 100% *f*T > 1x MIC with continuous infusions (CI) of 100–150 mg/kg/day (max 4/6 g) and 4-h infusions of 50 mg/kg (max 2 g), but > 90% PTA for 100% *f*T > 4x MIC was only achieved by 150 mg/kg CI. Decreased PTA was seen with less frequent dosing, shorter infusions, higher-dose CKRT, and higher residual kidney function.

**Conclusions:**

Our new CKRT-module was successfully added to an existing cefepime PK model for MCS in young patients on CKRT. When targeting 100% *f*T > 4xMIC or using higher-dose CKRT, CI would allow for higher PTA than intermittent dosing.

**Supplementary Information:**

The online version contains supplementary material available at 10.1186/s40560-024-00752-0.

## Background

Continuous kidney replacement therapy (CKRT) is frequently employed to support critically ill children with acute kidney injury (AKI), who have a high risk of mortality (36–50%) [1, 2]. CKRT is advantageous in critical illness because it allows for more precise volume control and greater hemodynamic stability than intermittent dialysis. Broad-spectrum antibiotics are often prescribed to patients receiving CKRT to treat suspected systemic infection or sepsis. Beta-lactam antibiotics are commonly prescribed for sepsis [3], with cefepime being the most frequently used in CKRT at our institution. Cefepime is susceptible to extracorporeal drug clearance (CL_EC_) because its low molecular weight [480.6 Daltons (Da)] and low degree of protein binding (~ 20%) allow it to pass through the filters used for CKRT, which have pore diameters up to 30,000 Da [4–6]. Despite this susceptibility to CL_EC_ via CKRT, the degree of, and variation in, cefepime CL_EC_ across varying patient characteristics and CKRT prescriptions are unknown.

Optimal cefepime dosing is difficult to estimate due to the complex combination of CKRT settings (e.g., filter size, blood flow rate, and effluent flow rate [*Q*_ef_], a measure of the dialysis dose provided) and patient characteristics (e.g., age, size, kidney and hepatic function, and fluid accumulation at any point in time), both of which affect pharmacokinetics (PK). Suboptimal dosing and consequent underexposure can lead to treatment failure, while overexposure has a risk of toxicity, specifically neurotoxicity for cefepime [7–10]. While published cefepime PK studies in adults receiving CKRT exist [11], there are only two small case series with four [12] and seven [13] children. Data from our institution [13] show the extent of CL_EC_ for cefepime may vary significantly, with CL_EC_ ranging from 31 to 74% of total patient cefepime CL (CL_tot_).

Recent reviews have found that PK studies are most often suboptimal for children receiving CKRT; they do not provide essential information, including residual kidney function, CKRT prescriptions, and patient volume status, needed for accurate dosing recommendations [14, 15]. Since beta-lactam antimicrobial effect depends on the percentage of time of a dosing interval that free drug concentration exceeds bacterial minimum inhibitory concentration (%*f*T > MIC) [16], some experts recommend administering cefepime in adults receiving CKRT as extended infusions to maximize probability of target attainment (PTA) [17, 18]. It is unknown if the same recommendation should apply to children receiving cefepime and CKRT.

In addition, fluid accumulation is common in patients with sepsis due to large volumes of fluid provided during the resuscitation phase and capillary leak from the release of inflammatory mediators during sepsis. When fluid accumulation is excessive, it can compromise critical end-organ function, and is termed fluid overload; fluid overload is known to be associated with worse outcomes for PICU patients [17] and is a common indication for initiation of CKRT [17, 18]. From a PK perspective, fluid overload can *increase* drugs’ volume of distribution, thereby *decreasing* peak concentrations and increasing half-lives [19], and must be considered when interpreting the PK of hydrophilic drugs such as beta-lactams.

Clinical trial simulation using Population PK (PopPK) models represents one method to address the knowledge gap regarding continuous infusions for children on CKRT, but no parametric PopPK models exist for cefepime for children receiving CKRT. Therefore, we aimed to develop and qualify a novel “module” representing CKRT that can be added to existing PopPK models for a given drug to account for CKRT and to perform Monte Carlo Simulations using realistic clinical covariates to estimate PTA (% of population achieving 100%*f*T > 1x or 4xMIC) of various cefepime dosing strategies in critically ill children receiving CKRT while considering the impact of (i) fluid accumulation, (ii) CKRT dose, and (iii) residual kidney function on PTA.

## Methods

### CKRT module and PK parameters

**Fig. 1:**
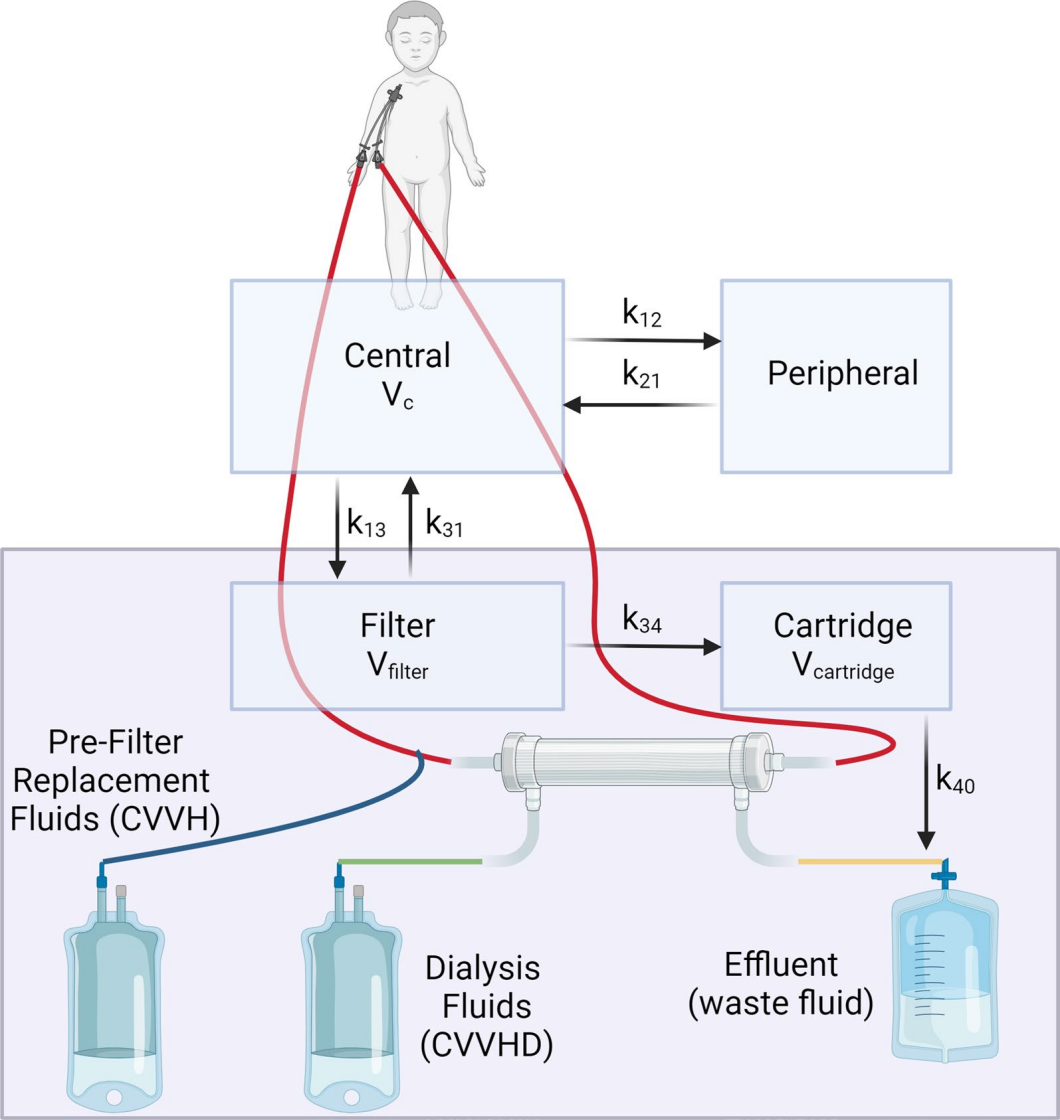
Schematic of CKRT model. CVVH, continuous veno-venous hemofiltration. CVVHD, continuous veno-venous hemodialysis. Mass transfer within this CKRT model includes PK rate constants representing transfer from the central compartment to the filter (*k*_13_), the filter to the central compartment (*k*_31_), the filter to the cartridge, or non-blood fluid space within the filter (*k*_34_), and then from the cartridge into the effluent (*k*_40_); see Table S1 for details. The purple shaded area represents the CKRT module connected to the central compartment. Created with BioRender.com

The PKPD-modeling software Edsim++ [20–22] (Mediware, Czech Republic) was used to develop a new module to account for CKRT. This module was extracted from a model of CKRT originally used for meropenem published by Nehus et al. [23] and Robatel et al. [24] and consists of two compartments representing the CKRT filter and dialysis fluid space (Fig. 1). The module allows for inputting individual components of the CKRT prescription and can be added to existing PopPK models of any drug. The rate constants and PK equations are described in Fig. 1 and Table S1.

To model the impact of fluid accumulation on target attainment, a scaling parameter was attached to the volume of distribution whereby the volume of the central compartment (*V*_c_) was increased by the percentage of fluid accumulation after linearly scaling to a weight of 70 kg as follows: *V*_c_ (scaling factor) = (WT/70 kg) * (1 + (FA/100)), where WT is weight, and FA is fluid accumulation. Given that only free, non-protein bound solutes are susceptible to CL_EC_, the module included a saturation coefficient (*S*_d_), or the expected ratio of concentration of a solute in the effluent compared with the concentration in the plasma. *S*_d_ is the hemodiafiltration equivalent of the sieving coefficient (*S*_c_) [25]. Small molecules such as urea that pass freely through dialysis filters have a *S*_d_ of 1.0, while large molecules such as albumin have a *S*_d_ of 0.6 Cefepime has ~ 20% plasma protein binding and literature-reported S_c_ and S_d_ have ranged from 0.64 to 0.83 [25], so an *S*_d_ of 0.8 was used.

The module was added to the PopPK model of cefepime in critically ill children published by Shoji et al. [26] In this model, clearance (L/h) is described by 0.395 * [− 0.09 + 1.09 * {1 − exp(− 0.00958 * PMA)}] * wt^0.75^ * (SCr/0.6)^−0.392^ and steady-state volume of distribution, Vss (L), is modeled as 0.46 * wt * (GA/30)^−0.548^, where PMA is post-menstrual age, wt is weight in kg, and SCr is serum creatinine in mg/dL. These equations were adapted via allometric scaling to 70 kg and *V*_1_ was estimated as Vss * FV1, where FV1 is the fraction of the volume attributed to *V*_1_ (*θ*_3_ in the final Shoji model). In the Shoji model, the standard deviation (SD) of CL was 0.1256 L/h (coefficient of variation, CV = 31.8%) and the SD for Vss was 0.09013 L (CV = 22.2%); both the point estimates and this associated variability were included in the model for MCS. The Shoji model was chosen as it, at the time of writing, is the only published parametric two-compartment model for cefepime pharmacokinetics in children.

### Qualification of the adapted cefepime poppk model with CKRT module with historically sampled patient data

To evaluate the performance of this CKRT module prior to using it for simulations, we used the module to estimate CKRT-attributable clearance in four patients who had scavenged opportunistic samples available while both on and off CKRT. We used the module to simulate concentration–time profiles across the time both on and off CKRT and to assess the goodness of fit of the model and estimated CL_EC_ and CL_tot_ to compare them to previously published values [13], which had been obtained by estimating CL while on CKRT and while off CKRT separately. Improvement in goodness of fit was determined by comparing the visual fit of the concentration–time profile to observed concentrations, quantifying the number of measured concentrations that fell within the 95% confidence interval for the concentration–time profile, and by comparing the bias (median prediction error, MdPE) and imprecision (median absolute prediction error, MdAPE) of the estimated concentration–time profile with and without inclusion of the CKRT module. Improvement in the majority of these parameters with inclusion of the CKRT module along with final bias of  ≤  ± 20% and imprecision of ≤ 35% were considered adequate goodness of fit.

### Patient populations for monte carlo simulations

Two sets of patients were generated for simulation. First, a virtual, “artificial” population was generated using random sampling from a uniform distribution of age with corresponding median weights and heights from 2021 U.S. Census data and partitioned into ages 2 to < 5, 5 to < 12, and 12 to < 25 years old (y.o.). An upper limit of 25 was chosen based on the demographics of patients admitted to our ICUs. Within each of these age categories, patients were assigned (1) either minimal residual kidney function (by assigning each patient a serum creatinine prior to CKRT initiation that would correspond to an estimated glomerular filtration rate, or eGFR of 5 mL/min/1.73 m^2^ per the bedside Schwartz formula [27]) or moderate residual kidney function (eGFR 30 mL/min/1.73 m^2^), (2) standard-dose CKRT [18, 28] with total effluent flow (*Q*_ef_) 2500 mL/hr/1.73 m^2^ or high-dose CKRT [29] with *Q*_ef_ 8000 mL/hr/1.73 m^2^, and (3) 0, 10, 20, or 30% fluid accumulation.

1000-fold Monte Carlo simulations were then performed using six cefepime dosing strategies: 150 mg/kg/day (max 6 g) as a continuous infusion (CI), 100 mg/kg/day (max 4 g) CI, 50 mg/kg/dose (max 2 g) every 8 h (q8h) as a 4-h extended infusion (EI), 50 mg/kg/dose (max 2 g) q8h as a 30-min standard infusion (SI), 50 mg/kg/dose q12h EI, and 50 mg/kg/dose q12h SI. PTA for 100%*f*T > 1x or 4xMIC using an MIC of 8 mg/L as the Clinical Laboratory Standards Institute breakpoint for *Pseudomonas aeruginosa* [30] was assessed for each of these regimens in the 5th–6th dosing interval to simulate steady-state target attainment. Estimated CL_EC_ and CL_tot_ were recorded for each set of simulations.

Since children requiring CKRT have important differences from the general pediatric population, a second set of simulations used real-world age, weight, height, fluid accumulation at CKRT initiation, and CKRT prescriptions from data collected from the prospective pediatric Continuous Renal Replacement Therapy (ppCRRT) database [18] partitioned into the same age categories as the census-derived artificial patient population. Since most children receiving CKRT are oligo-anuric, they were assigned an eGFR of 5 mL/min/1.73 m^2^. Fluid accumulation was calculated as (fluid intake since ICU admission in L − fluid output since ICU admission in L)/(ICU admit weight in kg). 1000-fold simulations of each of the above dosing regimens were performed and the same PK/PD data were assessed.

Regarding CKRT prescriptions, *Q*_b_ was set as 6 mL/kg/min up to 200 mL/min for virtual patients or actual *Q*_b_ for real-world patients. For artificial patients, *Q*_ef_ was assigned as standard or high dose as defined above; based on typical institutional clinical practice, *Q*_uf_ was set as 20% of total *Q*_ef_ for standard-dose CKRT and 12.5% of total *Q*_ef_ for high-dose CKRT. The remainder of *Q*_ef_ was split evenly between convective and dialytic modes as continuous veno-venous hemodiafiltration (CVVHDF) is commonly used in pediatric CKRT [31, 32]. Hemofiltration replacement fluids were simulated as pre-filter replacement. Actual delivered *Q*_ef_ was used for real-world patients. Patients 2 to < 5 years old were assigned to the 0.6 m^2^ ST60 filter (Baxter, Deerfield, IL) with *V*_filter_ and *V*_cartridge_ of 47 mL and 69 mL, patients 5 to < 12 years old were assigned the 1.0 m^2^ ST100 filter (Baxter) with *V*_filter_ and *V*_cartridge_ of 69 and 85 mL, and patients aged 12 to < 25 y.o. were assigned to the 1.4 m^2^ ST150 filter (Baxter) with *V*_filter_ and *V*_cartridge_ of 107 and 127 mL.

### Statistical analyses

For descriptive statistics of patient characteristics, means and standard deviations were reported as all variables were normally distributed. Simple linear regression was used to compare body surface area (BSA)-indexed *Q*_ef_ to CL_EC_/CL_tot_.

## Results

### Qualification of the adapted cefepime poppk model with CKRT module with historical sampled patient data

**Fig. 2:**
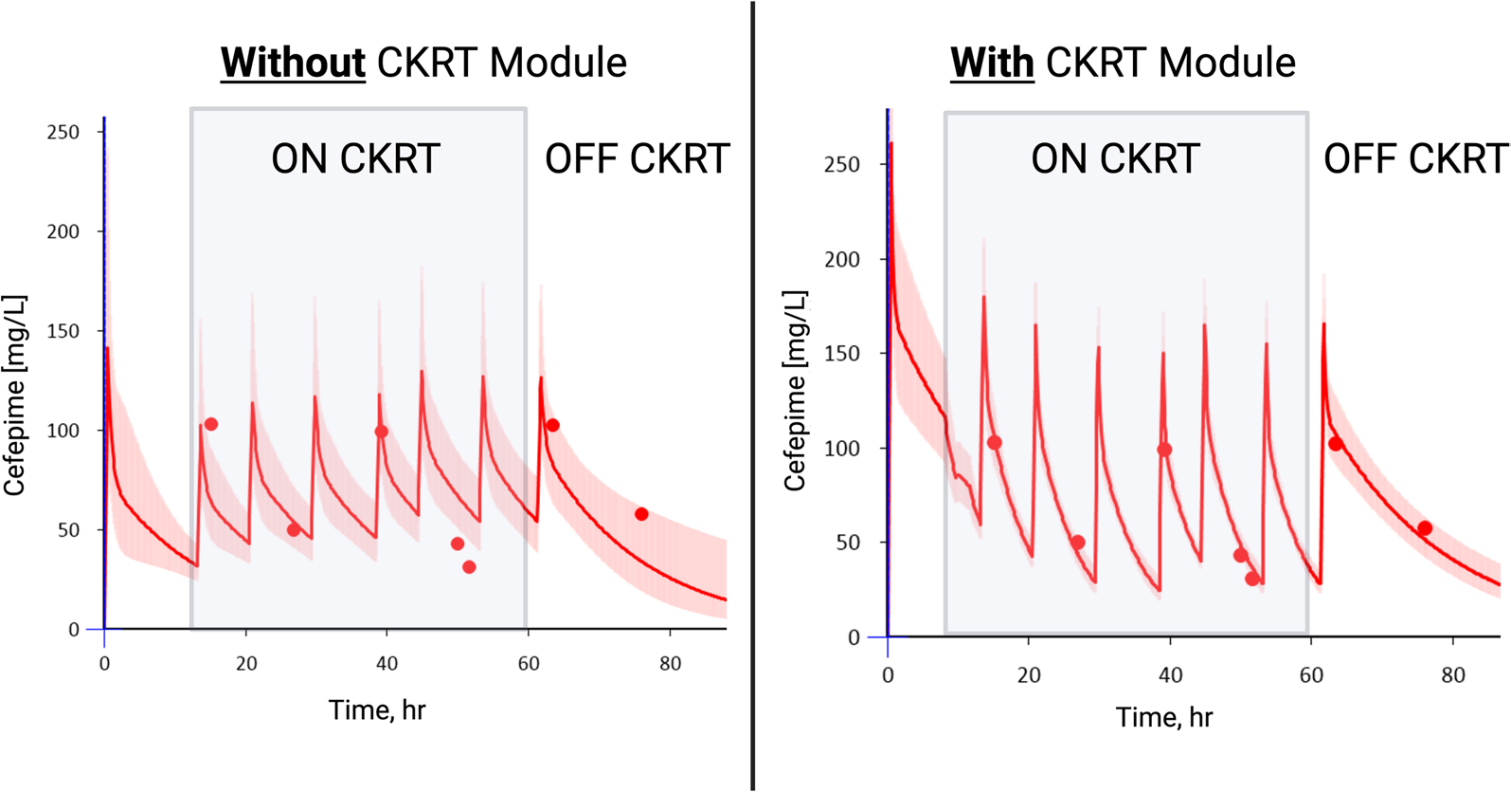
Comparison of model-informed precision dosing software-generated concentration–time profiles using observed cefepime plasma concentrations without (left panel) and with (right panel) inclusion of CKRT module for Patient 2. The closed circles are observed concentrations, the red solid line is the estimated concentration vs. time profile fitted to the observations and the red shaded area around the concentration–time profile is the 95th% percentile confidence interval. The blood flow rate was decreased from 200 mL/min to 100 mL/min at hour 12 of treatment, hence the inflection point in the predicted concentration–time profile at that point. Created with BioRender.com

Four patients had cefepime concentrations scavenged from residual blood samples available both while on and off CKRT. Inclusion of the CKRT module into the PopPK model allowed for seamless estimation of concentration–time profiles throughout both on- and off-CKRT periods (Fig. 2 and Figures S1–S3). For these four patients, previously [13] CL_EC_ was characterized by estimating total CL while off circuit and on circuit and subtracting off-circuit CL from total CL. The total CL was similar but the proportion of CL attributed to the extracorporeal circuit was higher estimated using the novel module (Table S2). A comparison of goodness-of-fit metrics with and without inclusion of the CKRT module is available in Table S3; inclusion of the CKRT module resulted in near universal improvement in markers of goodness of fit, which can be seen visually with the narrowing of the width of the 95% confidence interval in all four plots (Fig. 2 and Figs. S1–S3).

### Simulations with artificial patient population

**Fig. 3:**
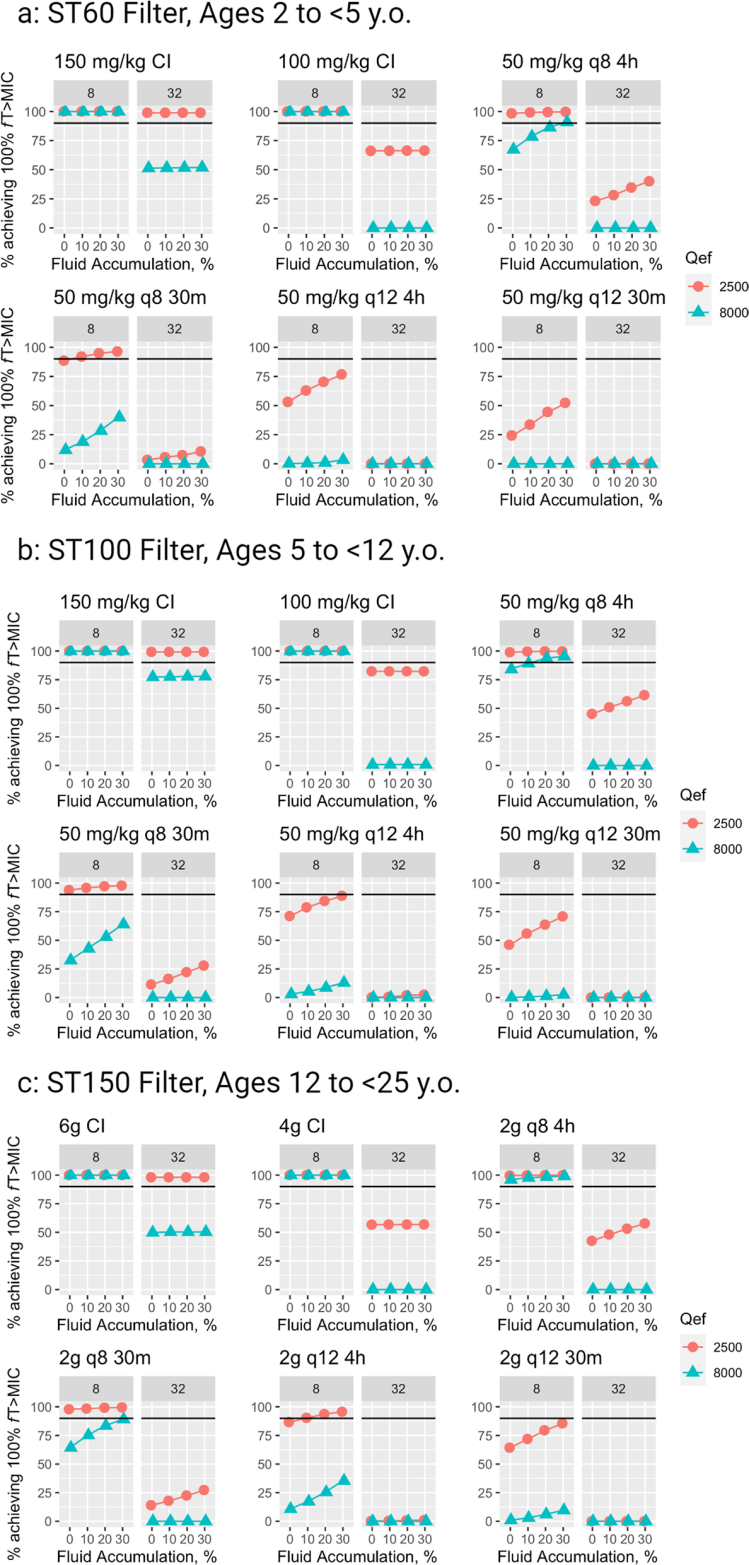
MCS from artificial patients with negligible residual kidney function (eGFR 5 mL/min/1.73 m^2^). CI, continuous infusion. q12 4 h, every 12 h as a 4-h infusion (EI, extended infusion). q12 30 m, every 8 h as a 30-min infusion (SI, standard infusion). q8 4 h, every 8 h as a 4-h infusion (EI). q8 30 m, every 8 h as a 30-min infusion (SI). Individual points represent the percentage of the 1000-fold simulated patients who achieved 100% *f*T > MIC, where 8 mg/L is 1xMIC and 32 mg/L is equivalent to 4xMIC. Black bars represent the PD target of 90% of population achieving 100% *f*T > MIC. Figure created with RStudio for Mac and BioRender

**Fig. 4:**
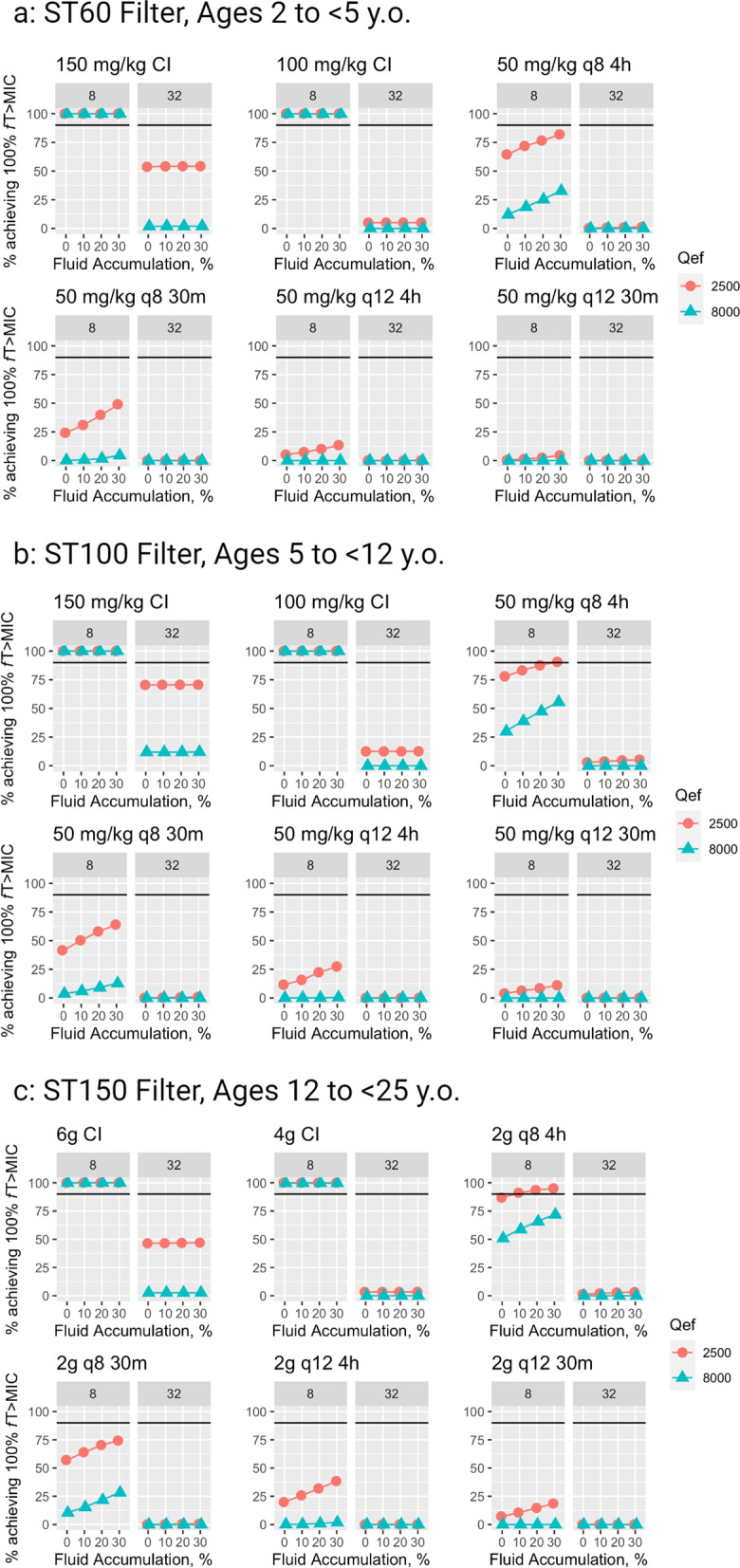
MCS from artificial patients with moderate residual kidney function (eGFR 30 mL/min/1.73 m^2^). CI, continuous infusion. q12 4 h, every 12 h as a 4-h infusion (EI, extended infusion). q12 30 m, every 8 h as a 30-min infusion (SI, standard infusion). q8 4 h, every 8 h as a 4-h infusion (EI). q8 30 m, every 8 h as a 30-min infusion (SI). Individual points represent the percentage of the 1000-fold simulated patients who achieved 100% *f*T > MIC where 8 mg/L is 1xMIC and 32 mg/L is equivalent to 4xMIC. Black bars represent the PD target of 90% of population achieving 100% *f*T > MIC. Figure created with RStudio for Mac and BioRender

Results from MCS using artificial patient data are presented in graphical format in Figs. 3, 4 and tabular format in Tables S4–S9. Across all age categories, increasing PTA was seen with lower-dose dialysis, longer cefepime infusion times, lower residual kidney function, and, for intermittent infusions only, higher degrees of fluid accumulation.

Figure 3 includes simulation results from all patients with minimal residual kidney function and demonstrates that CI maximized PTA across all age groups. When targeting 4x MIC, only 150 mg/kg/day CI was adequate to achieve > 90% PTA, and only when using standard-dose CKRT. No dosing regimen achieved 90% PTA for 100% *f*T > 4xMIC with high-dose CKRT. Older patients typically had higher PTA. Figure 4 depicts simulation results from all patients with moderate residual kidney function. PTA was globally lower in comparison with those with minimal residual kidney function.

The ratio of extracorporeal to total clearance (CL_EC_/CL_tot_) varied based on the degree of residual kidney function and the intensity of the CKRT prescription (Table S10), from ~ 25% in those with moderate residual kidney function and receiving standard-dose CKRT to ~ 63% in those with minimal residual kidney function and high-dose CKRT. Variation in *Q*_ef_ explained 99% of the variation in the CL_EC_/CL_tot_ ratio across all age groups in both eGFR categories.

### Comparisons with real-world data

Comparisons of patient characteristics and CKRT prescriptions from artificial versus real-world patients from the ppCRRT database are in Table S11. Mean ages, weights, % fluid accumulation, and CKRT *Q*_ef_ were similar, though there was greater variation in the degree of fluid accumulation and in CKRT *Q*_ef_ in the real-world patients and BSA-indexed *Q*_ef_ was lower in real-world patients aged 12 to < 25 y.o. In addition, body weight-indexed blood flow rate was lower in real-world patients. Comparison of mean simulation results using real-world patient data with results from the artificial patient population with eGFR 5 mL/min/1.73 m^2^ receiving standard-dose CKRT averaged across all fluid accumulation categories are presented in Table 1. Despite the above-noted differences in the underlying populations, there was universal concordance regarding potential dosing regimens recommended to achieve at least 90% PTA for 100% *f*T > 1x MIC and 100% *f*T > 4x MIC.

**Table 1:**
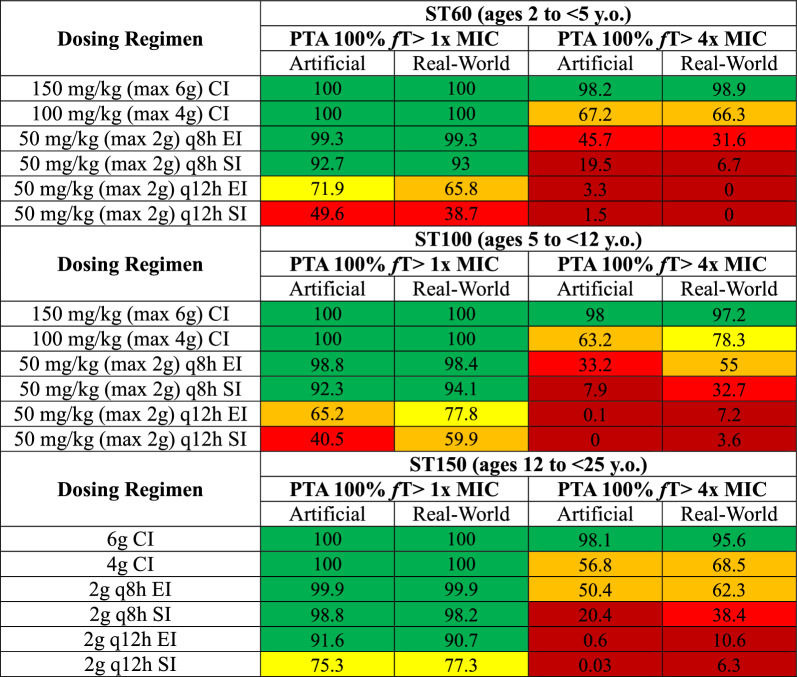
Comparison of PTA for artificial vs real-world patients with eGFR 5 mL/min/1.73 m^2^

CI, continuous infusion. EI, 4 h extended infusion. SI, 30-min standard infusion. Numbers in colored boxes represent the percent of 1000-fold simulated patients who achieved 100% *f*T > MIC. Results presented for artificial patients were with Q_ef_ 2500 mL/min/1.73 m^2^ averaged across all four fluid accumulation categories, given similar means in fluid accumulation between both groups as shown in this Table. Results for real-world patients were using actual Q_ef_ and fluid accumulation

## Discussion

Our results suggest that 4-h extended infusions or 24-h continuous infusions may be indicated to achieve stringent PD targets (i.e., 100% *f*T > 4x MIC) or when using high-intensity CKRT. It is notable that the recommended dosing regimens to achieve 90% PTA for 100% *f*T > 1x or 4x MIC were the same regardless of whether using artificial patients from U.S. census data or real-world data from the ppCRRT database, despite the differences in the patient populations and CKRT prescriptions (Table S11).

Large multi-center trials in adults (e.g., the RENAL and ATN trials) [33, 34] failed to show an improvement in outcomes for patients treated with higher-dose CKRT (*Q*_ef_ of 35–40 mL/kg/h) compared with “standard dose” CKRT (*Q*_ef_ of 20–25 mL/kg/h). One theorized reason for this failure to improve outcomes with higher-dose dialysis is that those receiving higher-intensity dialysis had increased CL of antimicrobials in the dialysis effluent without adjustment in the dose or frequency of antimicrobials. Our findings in this study are consistent with this hypothesis.

We found that PTA increased with increasing levels of fluid accumulation for intermittent infusions and are concordant with results published by Nehus et al. with meropenem [23]. When the volume of distribution of the central compartment increases due to fluid accumulation, the efficiency of elimination from the central compartment will diminish; if the clearance (measured in volume/time) remains the same while the concentration decreases, the rate of elimination will be lower, leading to a greater T > MIC. Steady-state PTA for continuous infusions was similar across fluid accumulation categories because with continuous infusions, the steady-state concentration is a function of the rate of infusion divided by the clearance, i.e., the volume of distribution is not relevant.

This paper employed Monte Carlo simulations, sometimes referred to as clinical trial simulations, as a strategy to test the potential impact of more variables than could be assessed in a typical clinical trial [35]. MCS use computer modeling to predict potential results based on the estimated probability of outcomes based on a given set of inputs. In this case, inputs into MCS included patient demographics (age and weight), the cefepime dosing regimen, degree of fluid overload, amount of residual kidney function, the CKRT prescription, and the target MIC threshold, for a total of 576 unique combinations of 1000 virtual patients. Since children receiving cefepime and CKRT is a rare event (for example, only approximately 10 patients per year at our high-volume children’s hospital receive both cefepime and CKRT), the use of MCS can help explore more potential combinations of patient, drug, and CKRT parameters than could feasibly be encountered in a routine clinical trial.

It is interesting to note that the simulations using real-world patient data often had modestly higher PTA than those using artificial patients. This may have been because the range of fluid accumulation and *Q*_ef_ was greater in the real-world patients. In addition, in the 12 to < 25 y.o. age group, delivered *Q*_ef_ was approximately 400 mL/h/1.73 m^2^ lower in real-world patients, which would similarly lead to a decrease in CL_EC_.

The PTA results from the 12 to < 25 y.o. patients for both artificial and real-world patients are concordant with findings from real-world data of adults receiving cefepime 2 g every 8 h as a 4 h EI while on CKRT from Philpott et al. [25] They found that this regimen resulted in uniform attainment of 100% *f*T > 1xMIC_8_ and near-uniform attainment of 100% *f*T > 4xMIC_8_ in adults receiving a CKRT prescription with *Q*_ef_ 30 mL/kg/h. The body weight-indexed Q_ef_ was ~ 40 mL/kg/h in the artificial patients presented herein, which could explain the lower PTA in these patients (50% vs 87.5% in Philpott’s report). In addition, in a simulation study of cefepime dosing in adults receiving CKRT by Al Shaer et al. [36], 2 g of cefepime administered as a 4-h infusion every 8 h was sufficient for nearly 100% probability of target attainment (PTA) of 100% *f*T > MIC at steady state when using an MIC of 8 mg/L and a Q_ef_ of 40 mL/kg/h, though PTA dropped to ~ 40% when targeting an MIC of 32 mg/L. For comparison, in our 12-to-25 y.o. patients with eGFR 5 mL/min/1.73 m^2^, comparable to an anuric state, with a *Q*_ef_ of 2500 mL/hr/1.73 m^2^ (~ 40 mL/kg/h when indexed to body weight rather than BSA), PTA for the same dosing regimen was 99.7% for *f*T > 1xMIC and 42.6% for *f*T > 4xMIC for an MIC of 8 mg/L. These findings suggest good performance of this model in adolescents and young adults despite its adaptation of a population PK model originally predominantly based on infants and young children.

Considering that cefepime is has predominantly renal elimination [4, 26, 37], it is notable that the ratio of CL_EC_/CL_tot_ averaged from 41 to 63% even in patients with minimal residual kidney function, indicating a significant proportion of non-renal and non-extracorporeal cefepime elimination. This finding may be due to the adaptation of the Shoji model [26], which did not include many patients with a creatinine > 1.0 mg/dL, potentially limiting its generalizability to patients with a low GFR. However, this range of CL_EC_/CL_tot_ is within the range in existing case reports of cefepime on CKRT [12, 13]. Moreover, even anuric patients have a decrease in cefepime concentrations over time [38], suggesting there is non-renal elimination of cefepime that has yet to be clearly described.

Strengths of this study include its consideration of multiple different dosing regimens across a wide spectrum of patient- and CKRT-related factors, including fluid accumulation, kidney function, and CKRT *Q*_ef_. Existing recommendations for drug dosing for children receiving CKRT do not take these factors into account. In addition, the similarity of PD target attainment results whether using artificial versus real-world patient data, along with the ability of the module to allow for estimation of a seamless concentration–time profile throughout both on- and off-circuit periods, suggests that this CKRT module may be useful in predicting cefepime PK/PD in children receiving CKRT. These simulations can form the basis of future studies to validate the performance of this model in real-world settings.

Limitations of this study include that the cefepime population PK model adapted to include CKRT was based predominantly on young children, the majority of whom did not have kidney dysfunction; therefore, it is possible that the model is over-estimating the amount of renal cefepime clearance in these patients. While a *S*_d_ of 0.8 was chosen, this study modeled hemodiafiltration (i.e., both convective and diffusive forms of solute removal), and it is known that hemodialysis is less efficient than hemofiltration in clearing “middle” molecules with a molecular weight > 500 Da, so these simulations may have overestimated hemodialysis-related clearance and thus CL_EC_/CL_tot_ [39]. This is of potentially greater concern with simulations using *Q*_ef_ of 8000 mL/h/1.73 m^2^ since hemodialysis-related clearance exhibits saturability at high dialysate flow rates [40]. In addition, since the threshold for cefepime-associated neurotoxicity in children is unknown, as reports of cefepime-associated neurotoxicity in children are limited to case reports, only two of which [9, 41] report any cefepime concentrations at all, we were unable to define dosing regimens that would minimize the likelihood of neurotoxicity. Finally, we performed these analyses on steady-state cefepime concentrations and did not investigate the impact of a loading dose, which is sometimes employed to achieve steady-state concentrations for CI more quickly [42, 43].

## Conclusions

This report of Monte Carlo simulations of cefepime dosing strategies using both artificially generated and real-world patient data showed concordant findings that continuous infusions may be beneficial to achieve stringent pharmacodynamic targets or when using high-dose CKRT. Our study demonstrates the robustness of using the CKRT module in combination with Monte Carlo simulations and is a potentially generalizable method for studying additional combinations of patient- and circuit-related factors across a range of medications. Future studies should validate the utility of this CKRT model in predicting extracorporeal clearance and target attainment in real-world pediatric patients receiving cefepime and CKRT.

## Supplementary information

Supplementary Material 1

## Data Availability

The datasets used and/or analyzed during the current study are available from the corresponding author on reasonable request.

## Abbreviations

AKI: Acute kidney injury
BSA: Body surface area
CCHMC: Cincinnati Children’s Hospital Medical Center
CI: Continuous infusion
CKRT: Continuous kidney replacement therapy
CL: Clearance
CL_EC_: Extracorporeal clearance
CL_tot_: Total clearance
CVVHDF: Continuous venovenous hemodiafiltration
Da: Dalton(s)
eGFR: Estimated glomerular filtration rate
EI: Extended infusion
FA: Fluid accumulation
MIC: Minimum inhibitory concentration
PD: Pharmacodynamic
PICU: Pediatric intensive care unit
PK: Pharmacokinetics
PMA: Postmenstrual age
PopPK: Population pharmacokinetic
ppCRRT: Prospective pediatric continuous renal replacement therapy
PTA: Probability of target attainment
q12h: Every 12 h
q8h: Every 8 h
*Q*_b_: Blood flow rate
*Q*_ef_: (Total) effluent flow rate
*Q*_uf_: Ultrafiltrate rate
*S*_c_: Sieving coefficient
SCr: Serum creatinine
*S*_d_: Saturation coefficient
SI: Standard infusion
*V*: Volume
WT: Weight
%fT > MIC: Time free concentrations exceed bacterial minimum inhibitory concentration
