# Supplementary material for: Monte Carlo simulations of cefepime in children receiving continuous kidney replacement therapy support continuous infusions for target attainment
Source: J Intensive Care. 2024 Oct 8;12:38. doi: 10.1186/s40560-024-00752-0 (PMC11459894; doi:10.1186/s40560-024-00752-0)
Supplement: Supplementary file 1 — Supplementary Material 1 [file 40560_2024_752_MOESM1_ESM.docx]

**Figure S1** Comparison of model-informed precision dosing software-generated concentration-time profiles using observed cefepime plasma concentrations without (left panel) and with (right panel) inclusion of CKRT module for Patient 1. The closed circles are observed concentrations, the red solid line is the estimated concentration vs. time profile fitted to the observations and the red shaded area around the concentration-time profile is the 95^th^% percentile confidence interval.


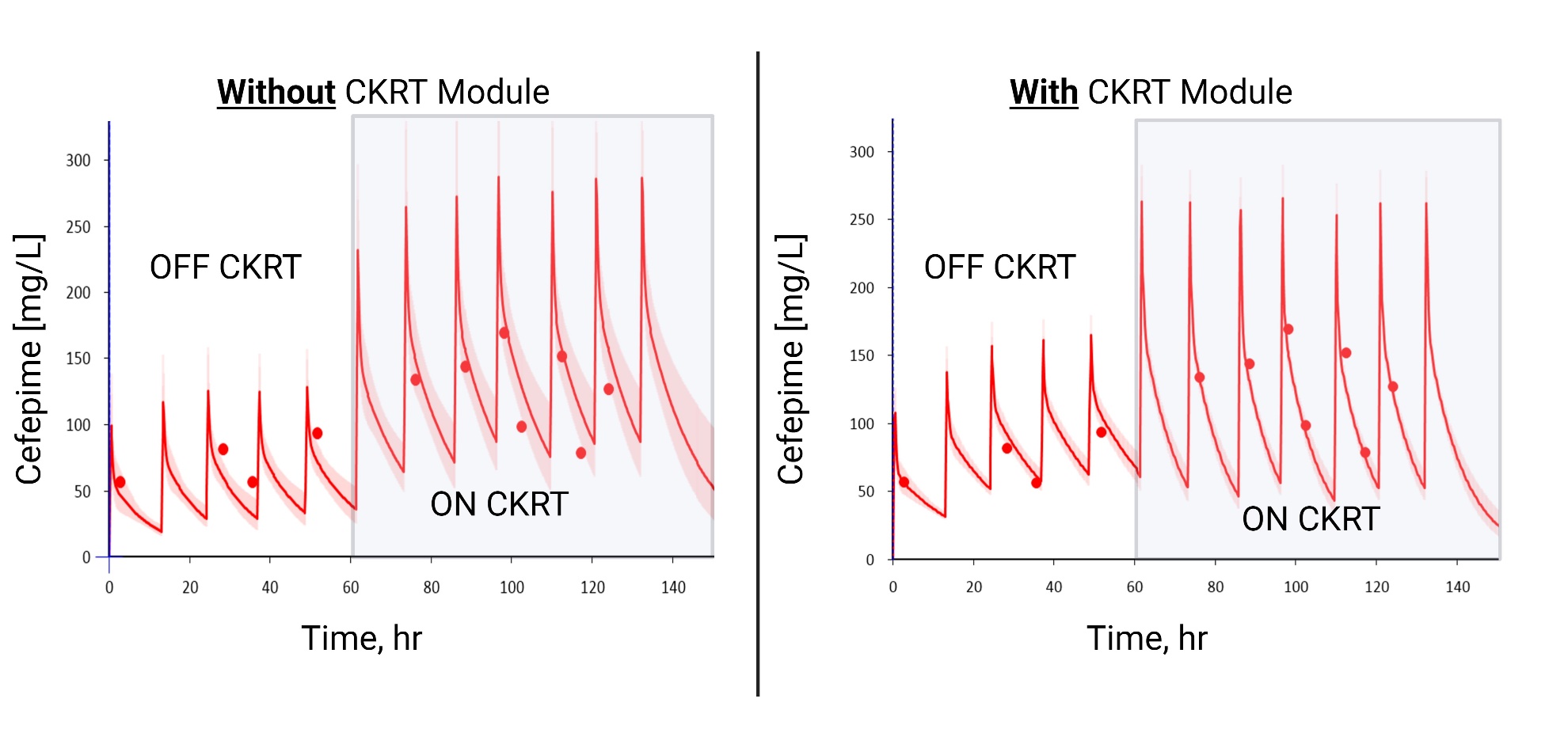


Created with BioRender.com.

**Figure S2** Comparison of model-informed precision dosing software-generated concentration-time profiles using observed cefepime plasma concentrations without (left panel) and with (right panel) inclusion of CKRT module for Patient 3. The closed circles are observed concentrations, the red solid line is the estimated concentration vs. time profile fitted to the observations and the red shaded area around the concentration-time profile is the 95^th^% percentile confidence interval.


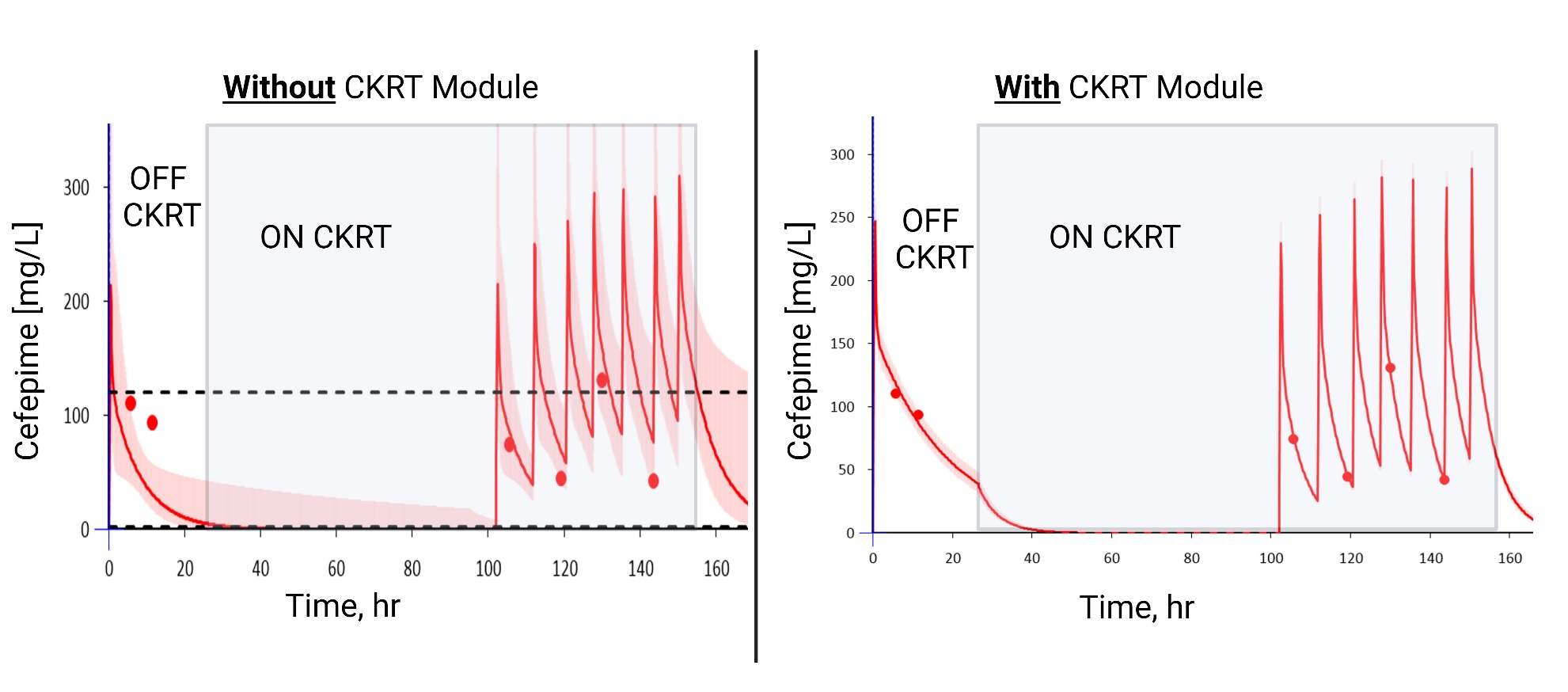


Created with BioRender.com.

**Figure S3** Comparison of model-informed precision dosing software-generated concentration-time profiles using observed cefepime plasma concentrations without (left panel) and with (right panel) inclusion of CKRT module for Patient 4. The closed circles are observed concentrations, the red solid line is the estimated concentration vs. time profile fitted to the observations and the red shaded area around the concentration-time profile is the 95^th^% percentile confidence interval.


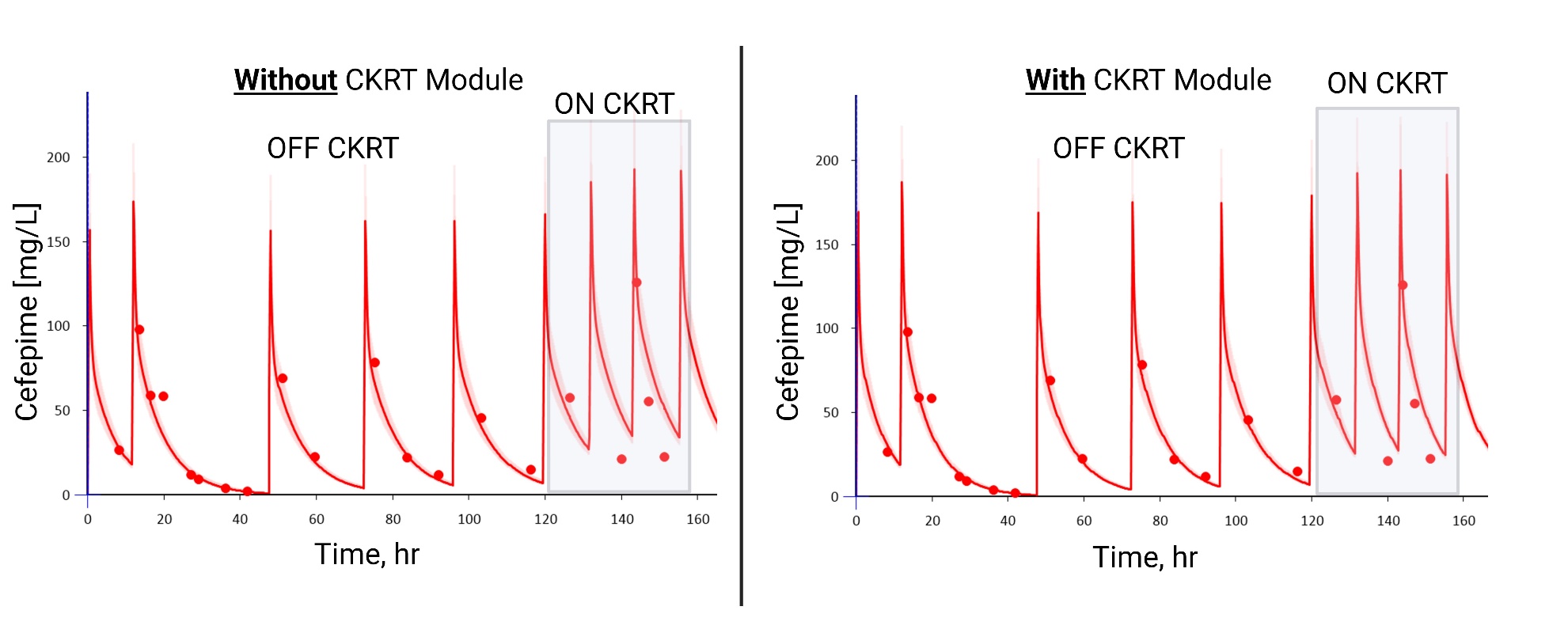


Created with BioRender.com.

**Table S1**: The rate constants can be described using the following equations:

| k_13_ = Q_plasma_/V_1_ | Rate constant for transfer of plasma from central compartment to filter. |
| --- | --- |
| k_31_ = (Q_plasma_ – Q_uf_)/V_filter_ | Rate constant for transfer of plasma from filter back to central compartment. |
| k_34_ = S_d_*(Q_ef_)/V_filter_ | Rate constant for transfer of plasma from filter to dialysis fluid space within filter. |
| k_40_ = (Q_ef_)/V_cartridge_ | Rate constant from transfer of plasma from dialysis fluid space to effluent. |

In these equations, V_1_ is the volume of the central compartment, V_filter_ is the volume of the blood compartment of the filter, and V_cartridge_ is the volume of the dialysate compartment of the filter. Q_plasma_ was defined as blood flow rate (Q_b_)*(1-Hct) assuming 30% Hct. Q_ef_ is a measure of total dialysis dose and is defined as Q_d_ (dialysis fluid flow rate) + Q_rep_ (replacement fluid flow rate) + Q_uf_ (net ultrafiltrate rate).

**Table S2**: Comparison of extracorporeal clearance (CL_EC_) vs total CL using prior method (estimating CL while on and off circuit separately) versus new method (using CKRT module).

| **Patient** | **CL_EC_ (L/h)** | **Total CL (L/h)** | **CL_EC_/**  **Total CL** |
| --- | --- | --- | --- |
| **1 - Prior** | 0.51 | 1.59 | 32% |
| **1 - New** | 1.19 | 1.89 | 63% |
| **2 - Prior** | 0.77 | 2.14 | 36% |
| **2 - New** | 1.85 | 2.46 | 75% |
| **3 - Prior** | 0.91 | 1.24 | 73% |
| **3 - New** | 1.06 | 1.37 | 77% |
| **4 - Prior** | 0.36 | 1.16 | 31% |
| **4 - New** | 0.69 | 1.34 | 51% |

**Table S3**: Goodness of fit metrics with and without inclusion of CKRT module.

| **Patient** | **# of concentrations within 95% CI of predicted concentration-time curve** | | **Bias (median prediction error)** | | **Precision (median absolute prediction error)** | |
| --- | --- | --- | --- | --- | --- | --- |
|  | **Without CKRT Module** | **With CKRT Module** | **Without CKRT Module** | **With CKRT Module** | **Without CKRT Module** | **With CKRT Module** |
| **1** | 6 of 11 | 10 of 11 | -7.5% | 2.5% | 23.3% | 8.4% |
| **2** | 4 of 7 | 7 of 7 | -2.2% | -4.9% | 38.1% | 11.3% |
| **3** | 4 of 6 | 6 of 6 | -17.3% | -1.8% | 47.2% | 2.4% |
| **4** | 15 of 19 | 16 of 19 | 5.0% | 3.9% | 14.1% | 12.9% |

CI, confidence interval.

**Table S4**: ST60, Ages 2 to <5 y.o., GFR 5 mL/min/1.73 m^2^

| **MIC, mg/L** | **Q_ef_, mL/hr/1.73 m^2^** | **% Fluid Accumulation** | **150 mg/kg/day (max 6g) continuous infusion** | **100 mg/kg/day (max 4g) continuous infusion** | **50 mg/kg (max 2g) q8h over 4h** | **50 mg/kg (max 2g) q8h over 30 min** | **50 mg/kg (max 2g) q12h over 4h** | **50 mg/kg (max 2g) q12h over 30 min** |
| --- | --- | --- | --- | --- | --- | --- | --- | --- |
| 8 | 2500 | 0 | 100 | 100 | 98.4 | 88.6 | 53.1 | 24.3 |
|  |  | 10 | 100 | 100 | 99.2 | 92 | 62.9 | 33.6 |
|  |  | 20 | 100 | 100 | 99.5 | 94.9 | 70.3 | 44.5 |
|  |  | 30 | 100 | 100 | 99.7 | 96.4 | 76.7 | 52.3 |
|  | 8000 | 0 | 100 | 100 | 67.3 | 11.8 | 0.2 | 0 |
|  |  | 10 | 100 | 100 | 78.3 | 18.8 | 0.4 | 0 |
|  |  | 20 | 100 | 100 | 86.3 | 28.3 | 1.0 | 0 |
|  |  | 30 | 100 | 100 | 90.8 | 39.9 | 3.3 | 0.1 |
| 32 | 2500 | 0 | 98.9 | 66.2 | 23.2 | 3.3 | 0 | 0 |
|  |  | 10 | 98.9 | 66.3 | 28.2 | 5.6 | 0 | 0 |
|  |  | 20 | 98.9 | 66.4 | 34.7 | 7.4 | 0 | 0 |
|  |  | 30 | 98.9 | 66.4 | 40.1 | 10.6 | 0 | 0 |
|  | 8000 | 0 | 51.3 | 0 | 0 | 0 | 0 | 0 |
|  |  | 10 | 51.5 | 0 | 0 | 0 | 0 | 0 |
|  |  | 20 | 51.7 | 0 | 0 | 0 | 0 | 0 |
|  |  | 30 | 51.9 | 0 | 0 | 0 | 0 | 0 |

h, hour. MIC, minimum inhibitory concentration. q, every. Q_ef_, total effluent flow (dialysis “dose”). Numbers in colored boxes represent the percent of 1000-fold simulated patients who achieved 100% *f*T > MIC.

**Table S5**: ST60, Ages 2 to <5 y.o., GFR 30 mL/min/1.73 m^2^

| **MIC, mg/L** | **Q_ef_, mL/hr/1.73 m^2^** | **% Fluid Accumulation** | **150 mg/kg/day (max 6g) continuous infusion** | **100 mg/kg/day (max 4g) continuous infusion** | **50 mg/kg (max 2g) q8h over 4h** | **50 mg/kg (max 2g) q8h over 30 min** | **50 mg/kg (max 2g) q12h over 4h** | **50 mg/kg (max 2g) q12h over 30 min** |
| --- | --- | --- | --- | --- | --- | --- | --- | --- |
| 8 | 2500 | 0 | 100 | 100 | 64.5 | 24.3 | 5.2 | 0.5 |
|  |  | 10 | 100 | 100 | 71.9 | 31 | 7.4 | 1.4 |
|  |  | 20 | 100 | 100 | 76.6 | 39.9 | 10.1 | 2.5 |
|  |  | 30 | 100 | 100 | 82.0 | 49.1 | 13.5 | 4.6 |
|  | 8000 | 0 | 100 | 99.9 | 12.0 | 0.2 | 0 | 0 |
|  |  | 10 | 100 | 99.9 | 18.6 | 0.6 | 0 | 0 |
|  |  | 20 | 100 | 99.9 | 25.2 | 1.7 | 0 | 0 |
|  |  | 30 | 100 | 99.9 | 32.7 | 4.5 | 0 | 0 |
| 32 | 2500 | 0 | 53.7 | 5 | 0.2 | 0 | 0 | 0 |
|  |  | 10 | 54.1 | 5 | 0.6 | 0 | 0 | 0 |
|  |  | 20 | 54.2 | 5 | 0.9 | 0 | 0 | 0 |
|  |  | 30 | 54.2 | 5 | 1.2 | 0 | 0 | 0 |
|  | 8000 | 0 | 1.8 | 0 | 0 | 0 | 0 | 0 |
|  |  | 10 | 1.9 | 0 | 0 | 0 | 0 | 0 |
|  |  | 20 | 1.9 | 0 | 0 | 0 | 0 | 0 |
|  |  | 30 | 1.9 | 0 | 0 | 0 | 0 | 0 |

h, hour. MIC, minimum inhibitory concentration. q, every. Q_ef_, total effluent flow (dialysis “dose”). Numbers in colored boxes represent the percent of 1000-fold simulated patients who achieved 100% *f*T > MIC.

**Table S6**: ST100, Ages 5 to <12 y.o., GFR 5 mL/min/1.73 m^2^

| **MIC, mg/L** | **Q_ef_, mL/hr/1.73 m^2^** | **% Fluid Accumulation** | **150 mg/kg /day (max 6g) continuous infusion** | **100 mg/kg/day (max 4g) continuous infusion** | **50 mg/kg (max 2g) q8h over 4h** | **50 mg/kg (max 2g) q8h over 30 min** | **50 mg/kg (max 2g) q12h over 4h** | **50 mg/kg (max 2g) q12h over 30 min** |
| --- | --- | --- | --- | --- | --- | --- | --- | --- |
| 8 | 2500 | 0 | 100 | 100 | 99 | 93.9 | 71.2 | 46.1 |
|  |  | 10 | 100 | 100 | 99.6 | 95.8 | 78.8 | 55.9 |
|  |  | 20 | 100 | 100 | 99.8 | 97.2 | 84.4 | 63.7 |
|  |  | 30 | 100 | 100 | 99.8 | 97.8 | 88.9 | 70.9 |
|  | 8000 | 0 | 100 | 100 | 84.1 | 32.5 | 2.9 | 0.1 |
|  |  | 10 | 100 | 100 | 89.2 | 42.7 | 5.2 | 0.5 |
|  |  | 20 | 100 | 100 | 93.6 | 53.0 | 8.5 | 1.2 |
|  |  | 30 | 100 | 100 | 95.3 | 64.0 | 12.9 | 2.5 |
| 32 | 2500 | 0 | 99.3 | 82.3 | 45.1 | 11.4 | 0.2 | 0 |
|  |  | 10 | 99.3 | 82.3 | 50.9 | 16.3 | 0.5 | 0 |
|  |  | 20 | 99.3 | 82.3 | 56.2 | 22.2 | 1.9 | 0.1 |
|  |  | 30 | 99.3 | 82.3 | 61.5 | 27.9 | 2.4 | 0.2 |
|  | 8000 | 0 | 77.3 | 0.7 | 0 | 0 | 0 | 0 |
|  |  | 10 | 77.4 | 0.8 | 0 | 0 | 0 | 0 |
|  |  | 20 | 77.8 | 0.8 | 0 | 0 | 0 | 0 |
|  |  | 30 | 77.9 | 0.8 | 0 | 0 | 0 | 0 |

h, hour. MIC, minimum inhibitory concentration. q, every. Q_ef_, total effluent flow (dialysis “dose”). Numbers in colored boxes represent the percent of 1000-fold simulated patients who achieved 100% *f*T > MIC.

**Table S7**: ST100, Ages 5 to <12 y.o., GFR 30 mL/min/1.73 m^2^

| **MIC, mg/L** | **Q_ef_, mL/hr/1.73 m^2^** | **% Fluid Accumulation** | **150 mg/kg (max 6g) continuous infusion** | **100 mg/kg (max 4g) continuous infusion** | **50 mg/kg (max 2g) q8h over 4h** | **50 mg/kg (max 2g) q8h over 30 min** | **50 mg/kg (max 2g) q12h over 4h** | **50 mg/kg (max 2g) q12h over 30 min** |
| --- | --- | --- | --- | --- | --- | --- | --- | --- |
| 8 | 2500 | 0 | 100 | 100 | 78.0 | 41.6 | 11.6 | 4.0 |
|  |  | 10 | 100 | 100 | 83.2 | 50.3 | 15.7 | 6.3 |
|  |  | 20 | 100 | 100 | 87.6 | 58.0 | 22.4 | 8.4 |
|  |  | 30 | 100 | 100 | 90.6 | 64.0 | 27.4 | 11.1 |
|  | 8000 | 0 | 100 | 100 | 29.9 | 3.6 | 0 | 0 |
|  |  | 10 | 100 | 100 | 38.9 | 5.9 | 0.1 | 0 |
|  |  | 20 | 100 | 100 | 47.4 | 9.0 | 0.1 | 0 |
|  |  | 30 | 100 | 100 | 55.5 | 12.7 | 0.4 | 0 |
| 32 | 2500 | 0 | 70.5 | 12.5 | 3.0 | 0.1 | 0 | 0 |
|  |  | 10 | 70.6 | 12.5 | 3.8 | 0.3 | 0 | 0 |
|  |  | 20 | 70.6 | 12.5 | 4.7 | 0.5 | 0 | 0 |
|  |  | 30 | 70.7 | 12.6 | 5.3 | 0.9 | 0 | 0 |
|  | 8000 | 0 | 11.9 | 0 | 0 | 0 | 0 | 0 |
|  |  | 10 | 11.9 | 0 | 0 | 0 | 0 | 0 |
|  |  | 20 | 11.9 | 0 | 0 | 0 | 0 | 0 |
|  |  | 30 | 12.0 | 0 | 0 | 0 | 0 | 0 |

h, hour. MIC, minimum inhibitory concentration. q, every. Q_ef_, total effluent flow (dialysis “dose”). Numbers in colored boxes represent the percent of 1000-fold simulated patients who achieved 100% *f*T > MIC.

**Table S8**: ST150, Ages 12 to <25 y.o., GFR 5 mL/min/1.73 m^2^

| **MIC, mg/L** | **Q_ef_, mL/hr/1.73 m^2^** | **% Fluid Accumulation** | **6g continuous infusion** | **4g continuous infusion** | **2g q8h over 4h** | **2g q8h over 30 min** | **2g q12h over 4h** | **2g q12h over 30 min** |
| --- | --- | --- | --- | --- | --- | --- | --- | --- |
| 8 | 2500 | 0 | 100 | 100 | 99.7 | 97.9 | 86.7 | 64.3 |
|  |  | 10 | 100 | 100 | 99.9 | 98.5 | 90.4 | 71.9 |
|  |  | 20 | 100 | 100 | 100 | 99.2 | 93.7 | 79.5 |
|  |  | 30 | 100 | 100 | 100 | 99.5 | 95.7 | 85.6 |
|  | 8000 | 0 | 100 | 100 | 96 | 64.3 | 10.7 | 0.9 |
|  |  | 10 | 100 | 100 | 97.7 | 75.1 | 17.2 | 3 |
|  |  | 20 | 100 | 100 | 98.4 | 83.5 | 25.4 | 6 |
|  |  | 30 | 100 | 100 | 99.1 | 89.1 | 35.4 | 9.6 |
| 32 | 2500 | 0 | 98.1 | 56.7 | 42.6 | 14 | 0.2 | 0 |
|  |  | 10 | 98.1 | 56.8 | 48.1 | 17.9 | 0.3 | 0 |
|  |  | 20 | 98.1 | 56.8 | 53.2 | 22.5 | 0.7 | 0 |
|  |  | 30 | 98.1 | 56.9 | 57.8 | 27.3 | 1 | 0.1 |
|  | 8000 | 0 | 49.8 | 0 | 0 | 0 | 0 | 0 |
|  |  | 10 | 50.3 | 0 | 0 | 0 | 0 | 0 |
|  |  | 20 | 50.3 | 0 | 0 | 0 | 0 | 0 |
|  |  | 30 | 50.3 | 0 | 0 | 0 | 0 | 0 |

h, hour. MIC, minimum inhibitory concentration. q, every. Q_ef_, total effluent flow (dialysis “dose”). Numbers in colored boxes represent the percent of 1000-fold simulated patients who achieved 100% *f*T > MIC.

**Table S9**: ST150, Ages 12 to <25 y.o., GFR 30 mL/min/1.73 m^2^

| **MIC, mg/L** | **Q_ef_, mL/hr/1.73 m^2^** | **% Fluid Accumulation** | **6g continuous infusion** | **4g continuous infusion** | **2g q8h over 4h** | **2g q8h over 30 min** | **2g q12h over 4h** | **2g q12h over 30 min** |
| --- | --- | --- | --- | --- | --- | --- | --- | --- |
| 8 | 2500 | 0 | 100 | 100 | 86.8 | 57.1 | 19.9 | 7.2 |
|  |  | 10 | 100 | 100 | 91.2 | 64.0 | 25.8 | 10.6 |
|  |  | 20 | 100 | 100 | 93.6 | 70.4 | 31.9 | 14.7 |
|  |  | 30 | 100 | 100 | 95.1 | 74.3 | 38.5 | 18.6 |
|  | 8000 | 0 | 100 | 99.7 | 50.8 | 10.4 | 0.1 | 0 |
|  |  | 10 | 100 | 99.7 | 58.7 | 15.3 | 0.2 | 0 |
|  |  | 20 | 100 | 99.7 | 65.7 | 21.7 | 0.9 | 0 |
|  |  | 30 | 100 | 99.7 | 71.8 | 28.2 | 1.8 | 0.1 |
| 32 | 2500 | 0 | 46.4 | 3.4 | 1.5 | 0.1 | 0 | 0 |
|  |  | 10 | 46.5 | 3.4 | 2.1 | 0.1 | 0 | 0 |
|  |  | 20 | 46.8 | 3.4 | 2.9 | 0.5 | 0 | 0 |
|  |  | 30 | 47.0 | 3.4 | 3.2 | 0.6 | 0 | 0 |
|  | 8000 | 0 | 2.7 | 0 | 0 | 0 | 0 | 0 |
|  |  | 10 | 2.7 | 0 | 0 | 0 | 0 | 0 |
|  |  | 20 | 2.7 | 0 | 0 | 0 | 0 | 0 |
|  |  | 30 | 2.7 | 0 | 0 | 0 | 0 | 0 |

h, hour. MIC, minimum inhibitory concentration. q, every. Q_ef_, total effluent flow (dialysis “dose”). Numbers in colored boxes represent the percent of 1000-fold simulated patients who achieved 100% *f*T > MIC.

**Table S10**: Comparing extracorporeal CL (CL_EC_) across the spectrum of kidney function and CKRT prescriptions. Results were obtained using 0% fluid accumulation and 50 mg/kg 4h infusion every 8 hours, though were only minimally different when different dosing regimens or fluid accumulation categories were chosen.

| **CKRT Dose,** mL/hr/1.73 m^2^ | **eGFR,** mL/min/1.73 m^2^ | **Filter / Age** | **CL_EC_, L/h (L/h/70 kg^0.75^)** | **CL_tot_, L/h (L/h/70 kg^0.75^)** | **CL_EC_/**  **CLtot** |
| --- | --- | --- | --- | --- | --- |
| 2500 | 5 | ST60, 2 to <5 y.o. | 0.659 (1.97) | 1.60 (4.76) | 0.413 |
|  |  | ST100, 5 to <12 y.o. | 1.06 (1.98) | 2.55 (4.78) | 0.415 |
|  |  | ST150, 12 to <25 y.o. | 1.69 (1.81) | 4.11 (4.41) | 0.411 |
|  | 30 | ST60, 2 to <5 y.o. | 0.647 (1.93) | 2.54 (7.58) | 0.255 |
|  |  | ST100, 5 to <12 y.o. | 1.05 (1.98) | 4.07 (7.62) | 0.259 |
|  |  | ST150, 12 to <25 y.o. | 1.683 (1.81) | 6.57 (7.05) | 0.256 |
| 8000 | 5 | ST60, 2 to <5 y.o. | 1.617 (4.82) | 2.56 (7.62) | 0.633 |
|  |  | ST100, 5 to <12 y.o. | 2.629 (4.93) | 4.12 (7.72) | 0.638 |
|  |  | ST150, 12 to <25 y.o. | 3.89 (4.18) | 6.31 (6.77) | 0.617 |
|  | 30 | ST60, 2 to <5 y.o. | 1.61 (4.79) | 3.50 (10.4) | 0.459 |
|  |  | ST100, 5 to <12 y.o. | 2.63 (4.92) | 5.64 (10.6) | 0.466 |
|  |  | ST150, 12 to <25 y.o. | 3.89 (4.17) | 8.77 (9.42) | 0.443 |

CKRT, continuous kidney replacement therapy. CL_EC_, extracorporeal clearance. CL_tot,_ total clearance. eGFR, estimated glomerular filtration rate. Numbers in parentheses represent allometrically scaled clearance values using a power of 0.75 to standardize to a patient weighing 70 kg for direct comparison of clearance values between age groups.

**Table S11**: Characteristics of artificial vs. real-world patients.

|  | **ST60 (ages 2 to <5 y.o.)** | |
| --- | --- | --- |
|  | **Artificial** | **Real-World** |
| **Age, mean** | 3.48 | 3.40 |
| **Sex, % female** | 48.9 | 32.5 |
| **Weight, mean (SD), kg** | 16.3 (1.5) | 15.1 (3.3) |
| **% fluid accumulation at time of CKRT initiation, mean (SD)** | 15 (11.2) | 18.4 (18.6) |
| **Q_b_, mean (SD), mL/min, (mL/kg/min)** | 98 (6) | 78 (5.3) |
| **CKRT Q_ef_, mean (SD), mL/hr/1.73 m^2^** | 2500 or 8000 (no SD as uniform) | 2494 (2139) |
|  | | |
|  | **ST100 (ages 5 to <12 y.o.)** | |
|  | **Artificial** | **Real-World** |
| **Age, mean** | 8.45 | 8.45 |
| **Sex, % female** | 48.9 | 48.1 |
| **Weight, mean (SD), kg** | 30.3 (8.7) | 32.9 (15.2) |
| **% fluid accumulation at time of CKRT initiation, mean (SD)** | 15 (11.2) | 15.1 (31.2) |
| **Q_b_, mean (SD), mL/min, (mL/kg/min)** | 167 (5.6) | 108 (3.8) |
| **CKRT Q_ef_, mean (SD), mL/hr/1.73 m^2^** | 2500 or 8000 (no SD as uniform) | 2477 (1343) |
|  | | |
|  | **ST150 (ages 12 to <25 y.o.)** | |
|  | **Artificial** | **Real-World** |
| **Age, mean** | 18.6 | 16.6 |
| **Sex, % female** | 49.2 | 42.3 |
| **Weight, mean (SD), kg** | 63.7 (6.6) | 63 (25.7) |
| **% fluid accumulation at time of CKRT initiation, mean (SD)** | 15 (11.2) | 10.9 (15.2) |
| **Q_b_, mean (SD), mL/min, (mL/kg/min)** | 200 (3.2) | 138 (2.6) |
| **CKRT Q_ef_, mean (SD), mL/hr/1.73 m^2^** | 2500 or 8000 (no SD as uniform) | 2074 (1175) |

CKRT, continuous kidney replacement therapy. Q_b_, blood flow rate. Q_ef_, total effluent flow rate. SD, standard deviation.
